# Importance of communication between health care professionals and forced migrant women during birth

**DOI:** 10.1093/eurpub/ckac131.458

**Published:** 2022-10-25

**Authors:** M Gaudion, M Engelhardt, J Kamhiye, T Borde

**Affiliations:** Alice Salomon Hochschule Berlin, Berlin, Germany

## Abstract

**Background:**

Communication and information are part of the Sexual and Reproductive Health and Rights (SRHR). Various studies show that successful communication between birthing person and health care professionals (HCP) has a positive impact on birth and lowers risk of traumatic birth experience for women. Since information and communication is a major challenge for both forced migrant women (FMW) and health workers during birth, we investigated experiences of both sides in qualitative study.

**Methods:**

Qualitative interviews were conducted with 7 maternal HCPs (midwifes, physicians, social workers) and with 7 FMW 1-9 months after the birth of their child in 3 regions in Germany. The refugee sample included new mothers from 6 countries of origins, 14 languages, and an average of three years living in Germany. The interviews were analyzed via framework analysis.

**Results:**

The majority of the interviewed FMW had no or little knowledge about SRHR. Good communication is one of the main factors allowing a safe and trustful environment with the birthing women. If verbal communication is not possible nonverbal communication helps to create and maintain a care relationship with the women is given. However, due to lack of staff, time and interpreters FMW with little German language proficiencies receive hardly any relevant information and had a poorer accompaniment during birth.

**Conclusions:**

To provide for equity and SRHRs in maternal health and care there is an urgent need for reliable professional interpretation and easily accessible information in relevant languages material about giving birth, medical possibilities, procedures and interventions. Additionally, further training on heterogeneous needs and life contexts is necessary, to improve professional care during birth in maternity wards. HCPs 1:1 support is strongly recommended.

**Key messages:**

• Information on SRHR and communication are a fundamental part of birth work and should be made possible for all women including FMW to prevent discrimination and traumatic birth experiences.

• If given, 1:1 support by HCPs during birth can comensate missing communication.

